# Intraocular iron injection induces oxidative stress followed by elements of geographic atrophy and sympathetic ophthalmia

**DOI:** 10.1111/acel.13490

**Published:** 2021-10-09

**Authors:** Yingrui Liu, Brent A. Bell, Ying Song, Hye J. Kim, Jacob K. Sterling, Benjamin J. Kim, Maura Poli, Michelle Guo, Kevin Zhang, Aditya Rao, Janet R. Sparrow, Guanfang Su, Joshua L. Dunaief

**Affiliations:** ^1^ Department of Ophthalmology The Second Hospital of Jilin University Changchun China; ^2^ F.M. Kirby Center for Molecular Ophthalmology Scheie Eye Institute Perelman School of Medicine at the University of Pennsylvania Philadelphia Pennsylvania USA; ^3^ Department of Ophthalmology Harkness Eye Institute Columbia University Medical Center New York New York USA; ^4^ Department of Ophthalmology Scheie Eye Institute University of Pennsylvania Philadelphia Pennsylvania USA; ^5^ Department of Molecular and Translational Medicine University of Brescia Brescia Italy; ^6^ Department of Molecular Life Science University of Pennsylvania Philadelphia Pennsylvania USA

**Keywords:** iron, lipid peroxidation, lipofuscin, oxidative stress, photoreceptor cells

## Abstract

Iron has been implicated in the pathogenesis of age‐related retinal diseases, including age‐related macular degeneration (AMD). Previous work showed that intravitreal (IVT) injection of iron induces acute photoreceptor death, lipid peroxidation, and autofluorescence (AF). Herein, we extend this work, finding surprising chronic features of the model: geographic atrophy and sympathetic ophthalmia. We provide new mechanistic insights derived from focal AF in the photoreceptors, quantification of bisretinoids, and localization of carboxyethyl pyrrole, an oxidized adduct of docosahexaenoic acid associated with AMD. In mice given IVT ferric ammonium citrate (FAC), RPE died in patches that slowly expanded at their borders, like human geographic atrophy. There was green AF in the photoreceptor ellipsoid, a mitochondria‐rich region, 4 h after injection, followed later by gold AF in rod outer segments, RPE and subretinal myeloid cells. The green AF signature is consistent with flavin adenine dinucleotide, while measured increases in the bisretinoid all‐*trans*‐retinal dimer are consistent with the gold AF. FAC induced formation carboxyethyl pyrrole accumulation first in photoreceptors, then in RPE and myeloid cells. Quantitative PCR on neural retina and RPE indicated antioxidant upregulation and inflammation. Unexpectedly, reminiscent of sympathetic ophthalmia, autofluorescent myeloid cells containing abundant iron infiltrated the saline‐injected fellow eyes only if the contralateral eye had received IVT FAC. These findings provide mechanistic insights into the potential toxicity caused by AMD‐associated retinal iron accumulation. The mouse model will be useful for testing antioxidants, iron chelators, ferroptosis inhibitors, anti‐inflammatory medications, and choroidal neovascularization inhibitors.

## Graphical abstract

Intravitreal injection of iron induces photoreceptor oxidative stress, resulting in increased autofluorescence, lipid peroxidation, and myeloid cell infiltration associated with retinal degeneration, geographic atrophy, and choroidal neovascularization.

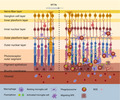

**Abbreviations**
8‐OHdG: 8‐hydroxy‐2′‐deoxyguanosine
AF: autofluorescence
AMD: age‐related macular degeneration
BAF: blue autofluorescence
CEP: carboxyethyl pyrrole
cSLO: confocal scanning laser ophthalmoscopy
FAC: ferric ammonium citrate
GFAP: glial fibrillary acidic protein
IRAF: infrared autofluorescence
IVT: intravitreal
L‐Ft: light ferritin
MDA: malondialdehyde
OCT: optical coherence tomography
RPE: retinal pigment epithelium
TUNEL: Terminal deoxynucleotidyl transferase dUTP nick end labeling
Z01: zonula occludens 1

## 1 INTRODUCTION

Iron is widely distributed throughout the human retina and plays an essential role in physiological processes, including respiration and the visual cycle (Picard et al., 2020). Yet, when dysregulated, it can produce the most reactive of the free radicals: hydroxyl radical (Wong et al., 2007).

Iron has been shown to cause or exacerbate multiple retinal diseases. For example, iron accumulation has been reported in the retinas of patients with age‐related macular degeneration (AMD) (Biesemeier et al., 2015; Hahn et al., 2003). Ocular siderosis due to an iron‐containing intraocular foreign body causes rapid retinal degeneration, as photoreceptors are especially sensitive to iron toxicity, most likely due to their high concentration of oxygen and easily oxidized polyunsaturated fatty acids (SanGiovanni & Chew, 2005). Retinal iron accumulation also causes retinal degeneration in mice with hereditary retinal iron overload (Hadziahmetovic et al., 2008, 2011, 2011; Hahn et al., 2004), which is protected by systemic treatment with the iron chelator deferiprone (Song et al., 2014).

Previous studies focusing on acute effects reported that intravitreal (IVT) iron injection leads to lipid peroxidation, photoreceptor degeneration, and increased retinal pigment epithelium (RPE) lipofuscin (Dunaief, 2006; Rogers et al., 2007; Shu et al., 2020). Anderson et al. (1984) reported that frogs with IVT iron injection had decreased polyunsaturated fatty acids and increased lipid hydroperoxides in isolated rod outer segments. Hiramitsu et al. (1976) reported photoreceptor degeneration induced by IVT injections of linoleic acid hydroperoxide in rabbits. Ferroptosis, an iron‐dependent programmed cell death pathway, is accompanied by lipid peroxide accumulation (Yang & Stockwell, 2016) and occurs in the sodium iodate retinal degeneration model (Tang et al., 2021). Taken together, iron may induce retinal degeneration via peroxidation of polyunsaturated fatty acids.

Iron‐induced retinal degeneration is also associated with bisretinoid oxidation (Ueda et al., 2018). Bisretinoid lipofuscin comprises intra‐lysosomal autofluorescent material deposited in the RPE cells. RPE lipofuscin accumulates with age (Wing et al., 1978), and in several retinal diseases including recessive Stargardt disease, where it accumulates in abundance (Delori et al., 1995), and AMD (Delori et al., 2000; Dorey et al., 1989; Holz et al., 1999; Lois et al., 2002; Sparrow & Boulton, 2005). Intracellular iron has been shown to catalyze bisretinoid A2E oxidation and degradation, eliciting cellular damage by releasing aldehyde‐ and dicarbonyl‐bearing fragments. Iron chelation by deferiprone decreased A2E oxidative degradation and protected against cell death (Ueda et al., 2018).

Previously, we studied the acute effects of IVT ferrous sulfate injection, which induced photoreceptor degeneration with increased oxidative stress and lipid peroxidation (Shu et al., 2020). Here, we used ferric ammonium citrate (FAC), which is commonly used experimentally to load cells with iron, is more stable than ferrous sulfate, and yields more reproducible amounts of retinal damage. We extended prior studies with IVT iron by elucidating novel iron‐induced changes in bisretinoids and production of carboxyethyl pyrrole, a specific docosahexanoic acid oxidation product implicated in AMD. We also found, for the first time, progressive geographic atrophy of the RPE, and, unexpectedly, iron‐induced myeloid cells infiltrating the saline‐injected control eyes of mice that had FAC injections in their fellow eyes.

## 2 RESULTS

### 2.1 FAC injection induced acute pan‐retinal AF and photoreceptor degeneration

To test our hypothesis that FAC, unlike ferric sulfate, would cause retinal toxicity because of FAC's higher solubility, we measured total soluble iron and ferrous iron in phosphate‐buffered saline at pH 7.4 with vitreous levels of ascorbate (2 mM). The solution with 50 μM FAC (monoferric) added had 42.5 ± 3.5 μM soluble iron with 31.2 ± 1.4 μM ferrous iron. In contrast, the solution with 25 μM ferric sulfate (diferric) added had only 33.0 ± 0.7 μM soluble iron with 13.0 ± 1.6 μM ferrous iron.

**FIGURE 1:**
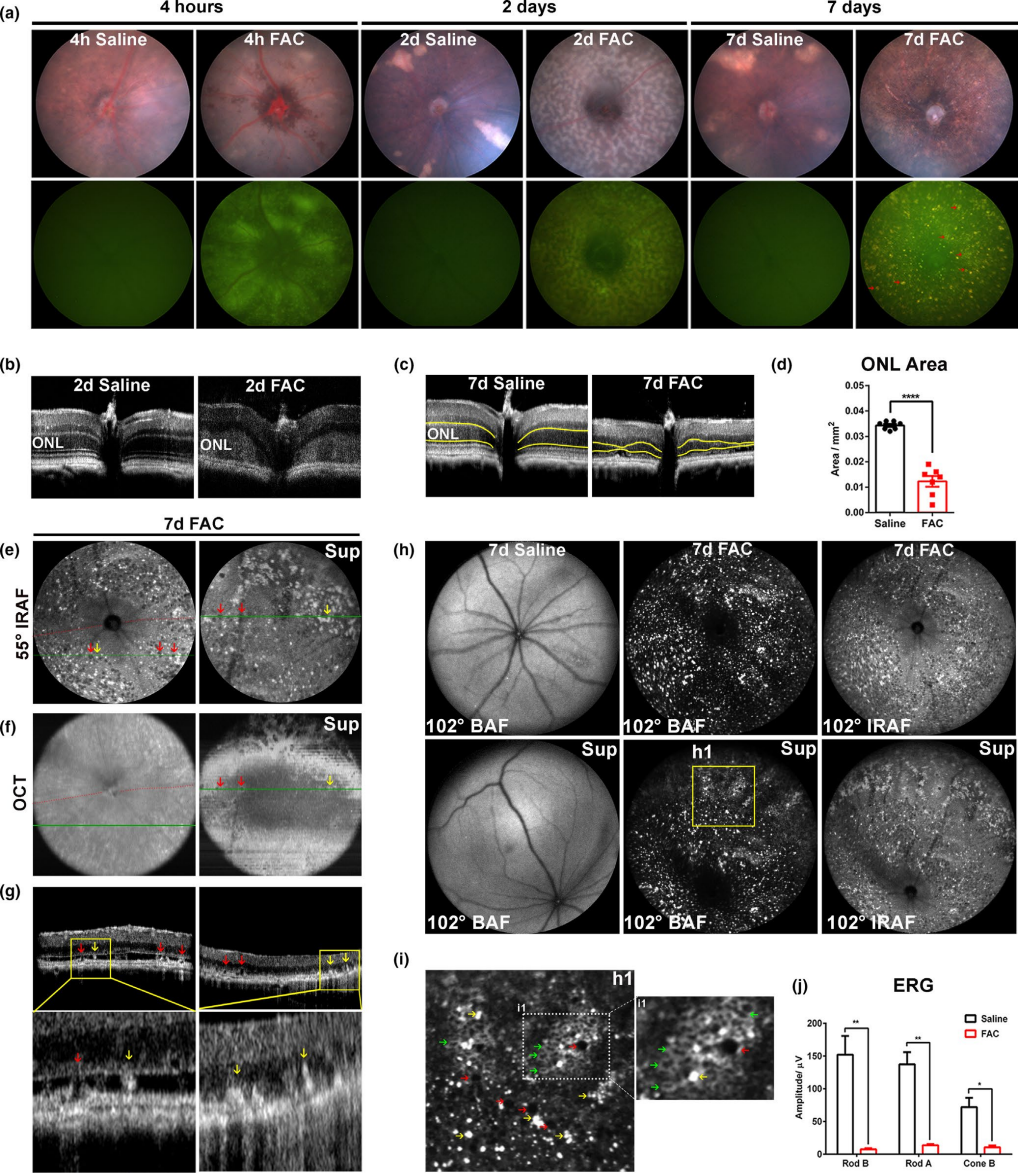
IVT ferric ammonium citrate (FAC) induced pan‐retinal autofluorescence (AF) and photoreceptor degeneration. Color fundus photographs and fundus AF photographs were obtained at 4 h, 2 days, and 7 days after FAC or control saline injection (a). Horizontal optical coherence tomography scans through optic nerve acquired at 2 days (b) and 7 days (c) after injection. Outer nuclear layer area in horizontal optical coherence tomography scans (c, yellow line) was quantified using ImageJ (d). 55° wide field infrared AF cSLO images were acquired from the central retina and superior peripheral retina 7 days after FAC injection (e). Optical coherence tomography images acquired from the same mouse and time points as in panel e are shown in panel f. The positions of selected optical coherence tomography scans (g) were indicated in the optical coherence tomography fundus view (f, green line). Long posterior ciliary arteries (red dotted line) were used as a natural landmark for the orientation of images. 102° ultra‐wide field blue‐AF and infrared AF cSLO images were acquired at 7 days after FAC and saline injection (h). A magnified image of the 102° blue AF cSLO image (h1, yellow box) is shown (i). ERG was conducted at 2 weeks after injection, (j). For each set of images, representative images were chosen from N = 8–10. All yellow arrows indicate hyper‐autofluorescent spots, red arrows indicate hypo‐autofluorescent spots, and green arrows indicate hyper‐autofluorescent binuclear RPEs. OCT, optical coherence tomography; ONL, outer nuclear layer; BAF, blue AF; IRAF, infrared AF; Sup, superior retina. Error bars indicate mean ± SEM. *p < 0.05, **p < 0.01, N = 3‐5/ group for ONL thickness measurement

Following IVT FAC injection, in vivo imaging was used to visualize retinal phenotypes. FAC injection caused pan‐retinal hypopigmentation detected with color fundus photography and diffuse green emission in the AF photographs at 4 h after injection. By 2 days after FAC injection, the pan‐retinal hypopigmentation and AF became more pisciform and included some red AF emission. At 7 days after FAC injection, diffusely distributed hypopigmented spots were prominent on color fundus photography, and a mixture of gold or green‐emitting hexagonal RPE cells, as well as gold‐emitting myeloid cells were visible on fundus AF (Figure 1a and Figure 4 for histologic verification). A few hypopigmented patches appeared in the retina at both 2 days and 7 days after control saline injections, but these were larger sectoral lesions rather than small spots, and lacked AF, so they were quite distinct from FAC‐induced lesions (Figure 1a). In optical coherence tomography scans, the outer retina became hyper‐reflective at 2 days after FAC injection (Figure 1b), and the outer nuclear layer was markedly thinned by 7 days (Figure 1c). The outer nuclear area (yellow lines) was significantly decreased after FAC injection (Figure 1d). To evaluate fundus AF, scanning laser ophthalmoscopy short‐wavelength (blue) AF and near‐infrared AF imaging were performed. Wide‐field near‐infrared AF images (Figure 1e) and optical coherence tomography volume intensity projections (Figure 1f) from the same eye at 7 days were selected to display the FAC‐induced changes. The near‐infrared AF images displayed intense pan‐retinal hyper‐autofluorescent (yellow arrows) and hypo‐autofluorescent spots (red arrows), corresponding to hyper‐reflective subretinal foci (yellow arrows) and hypo‐reflective spots (red arrows), respectively, on optical coherence tomography scans (Figure 1g). Atrophic or amelanotic RPE cells have been reported to be hypo‐autofluorescent in infrared AF imaging (Duncker et al., 2014; Sunness et al., 2020). RPE atrophy was present in optical coherence tomography scans in the superior retina after FAC injection (right side of scan, yellow box). 102° ultra‐wide field blue and infrared AF images showed FAC induced pan‐retinal hyper‐ and hypo‐autofluorescent spots, most likely representing myeloid cells (hyper‐AF) and RPE atrophy (hypo‐AF) (Figure 1h). An enlarged blue AF image from the superior retina (Figure 1h, yellow box) displays clustered hyper‐autofluorescent spots, representing RPE, myeloid cells, and photoreceptor layer undulations (yellow arrows), hyper‐autofluorescent binuclear RPEs (green arrows), and hypo‐autofluorescent foci of RPE atrophy (red arrows) (Figure 1i). FAC injection caused significant decreases in electroretinogram rod b‐wave, rod a‐wave, and cone‐b wave amplitudes compared with saline‐injected eyes, consistent with the FAC‐induced photoreceptor degeneration (*p < 0.05, **p < 0.01, Figure 1j).

### 2.2 IVT FAC caused photoreceptor and RPE AF and an increase in the bisretinoid all‐trans‐retinal dimer

**FIGURE 2:**
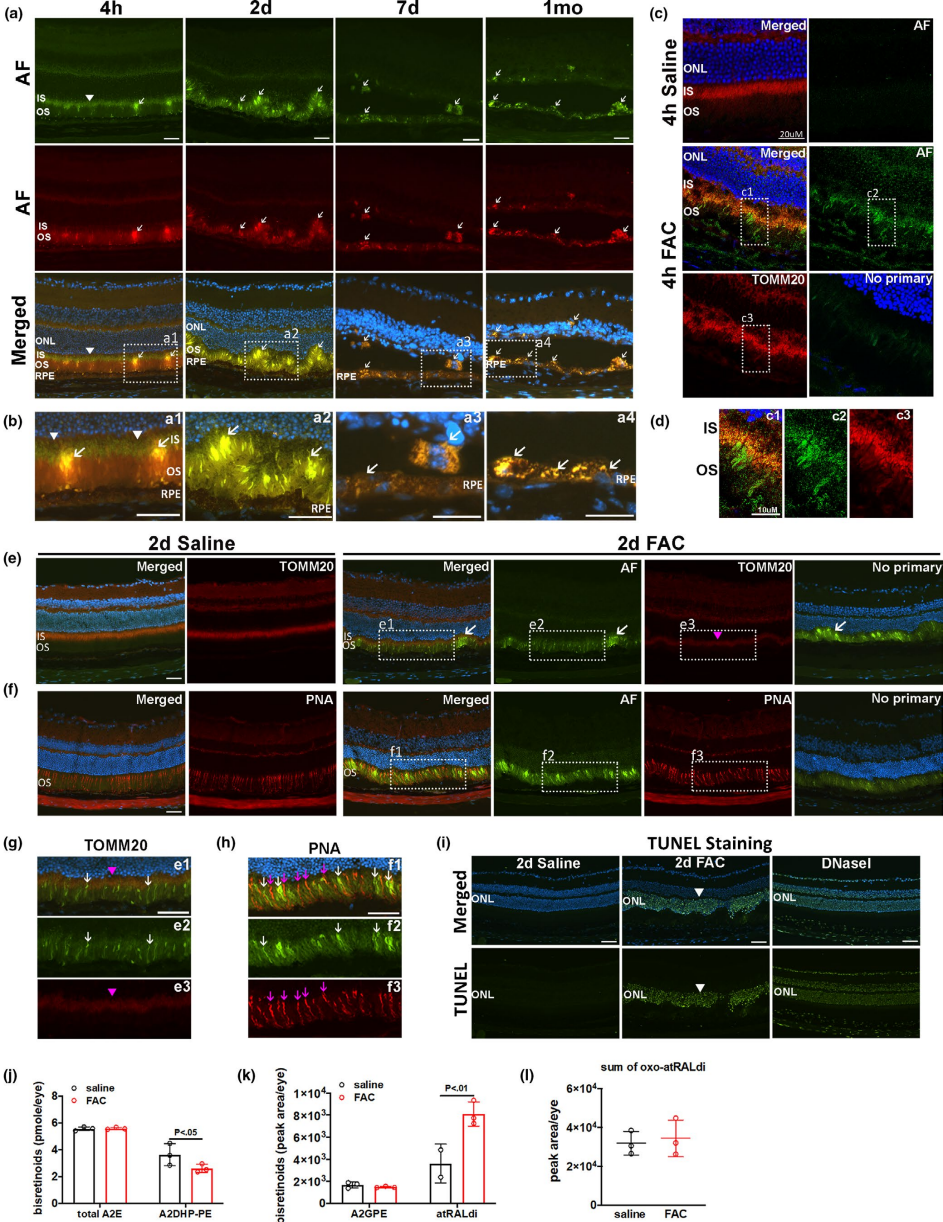
Ferric ammonium citrate (FAC) induced autofluorescence (AF) in mitochondria, rod outer segments, and atRALdi accumulation. Epifluorescence photomicrographs (unless indicated as confocal) of AF on cryosections obtained after FAC injection (a). Enlarged images for AF (b). Confocal imaging of green‐emitting AF and immunolabeling for TOMM20 (mitochondrial marker) were performed on cryosections prepared at 4 h after FAC and saline injection (c). Enlarged images of AF and TOMM20 (d). Green‐emitting AF and TOMM20 labeling was performed on cryosections at 2 days after injection (e). Green‐emitting AF and peanut agglutinin labeling for cones performed on cryosections at 2 days after injection (f). Enlarged images for green‐emitting AF and labeling with TOMM20 (g). Enlarged images for green‐emitting AF and labeling with peanut agglutinin (h). TUNEL labeling was conducted on cryosections prepared on 2 days after injection (i). DNase I was used as the positive control for TUNEL labeling. Quantification of bisretinoids by high pressure liquid chromatography from whole eyes at 7 days after FAC and saline injection (j‐l). All white arrows indicate AF, magenta triangles indicate immunolabeling for TOMM20, and magenta arrows indicate labeling with peanut agglutinin. White triangles indicate AF in a. White triangles indicate positive TUNEL labeling in i. ONL, outer nuclear layer; IS, inner segment; OS, outer segment; RPE, retinal pigmented epithelium; PNA, peanut agglutinin. Error bars indicate mean ± SEM. *p < 0.05, **p < 0.01, N = 3‐5/group for bisretinoid measurements. Representative immunolabeling images are shown from N = 3 mice per group. Scale bar: 50 µm

Cryosections obtained from FAC‐ and saline‐injected eyes were used to identify and localize FAC‐induced autofluorescent cells. FAC induced AF at 4 h, 2 days, and 7 days and 1 month after injection (Figure 2a and b, white arrows). By 4 h after FAC injection, green‐emitting AF was present in photoreceptor inner segments, and broad wavelength‐emitting (both green and red) AF was apparent in some photoreceptor outer segments. To localize the FAC‐induced AF in the photoreceptor inner segments, immunolabeling with anti‐TOMM20 was used to label photoreceptor mitochondria and imaged by confocal microscopy (Figure 2c,d). At 4 h after FAC injection, the green AF in photoreceptor inner segments co‐localized with the TOMM20 labeling, suggesting FAC induced green‐emitting AF (possibly flavin adenine dinucleotide, see Discussion) in the mitochondria (Figure 2d). By 2 days after injection, the photoreceptor inner segment AF diminished but photoreceptor outer segment AF remained (Figure 2e,g). Undulating folds of the photoreceptors were apparent, most likely producing some of the autofluorescent spots or flecks seen with in vivo imaging. By 7 days and 1 month, FAC had induced severe loss of photoreceptors with very thin outer nuclear layer and red/green‐emitting AF in the RPE layer. Labeling with peanut agglutinin was used to visualize cone photoreceptors. The AF (white arrows) did not co‐localize well with the cone photoreceptors (magenta arrows), suggesting FAC induced AF was emitted from rod photoreceptor outer segments (Figure 2h,f).

TUNEL labeling was conducted on cryosections prepared 2 days after FAC or saline injection to evaluate retinal cell death. Many photoreceptor nuclei were TUNEL positive after FAC injection (Figure 2i).

Since bisretinoids constitute autofluorescent RPE lipofuscin (Sparrow et al., 2012), we assessed bisretinoid levels in whole globes at 7 days. Total A2E and A2‐GPE showed no difference compared to saline, A2DHP‐PE was significantly reduced, but all‐trans retinal dimer (atRALdi) was significantly increased (Figure 2j,k). The level of oxidized atRALdi showed no difference compared to the saline controls (Figure 2l).

### 2.3 IVT FAC induced iron accumulation in Müller glia and myeloid cells, and the formation of lipid peroxidation products

**FIGURE 3:**
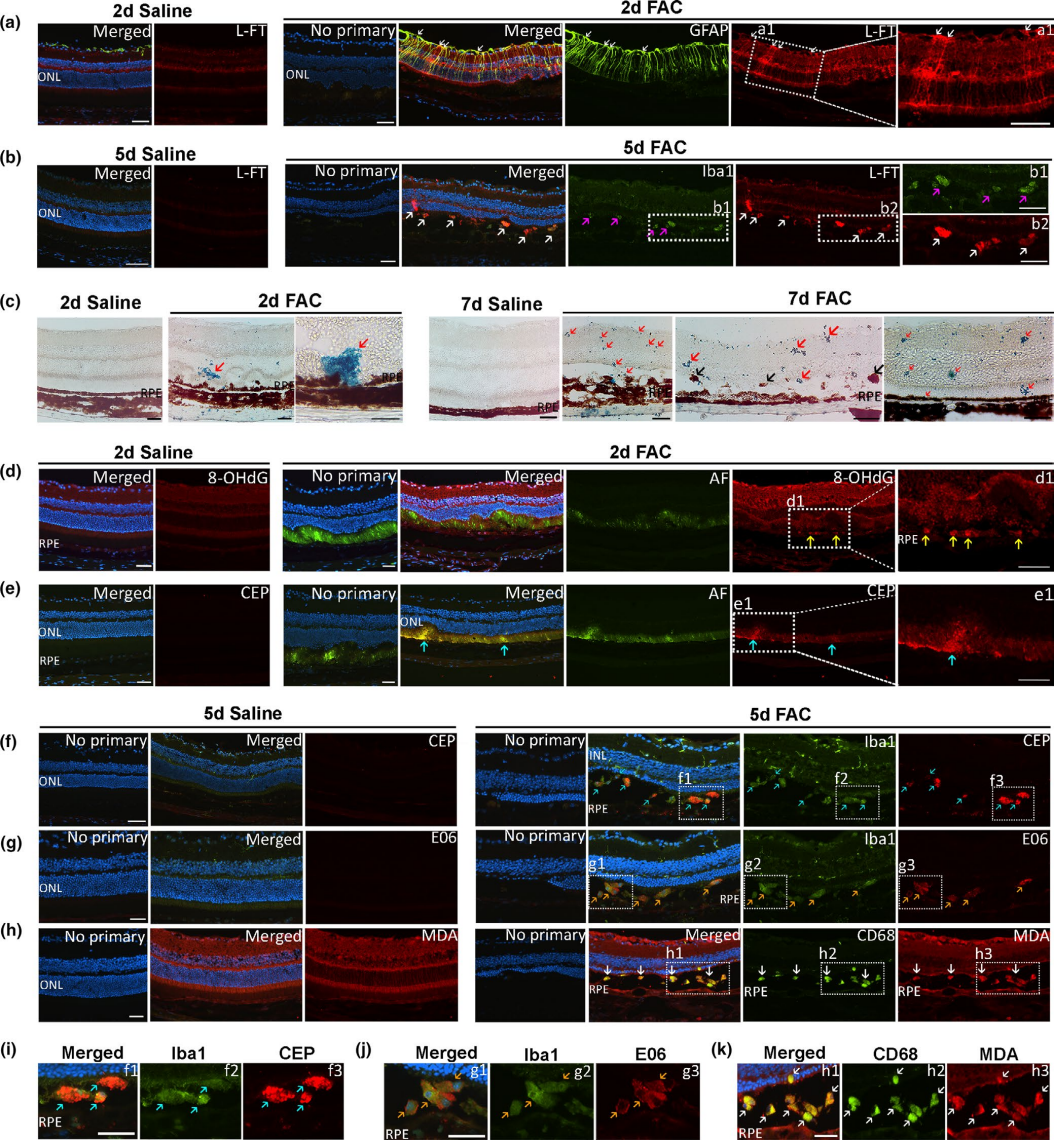
Ferric ammonium citrate (FAC) induced retinal iron accumulation and lipid peroxidation products. Epifluorescence photomicrographs of co‐labeling for GFAP and L‐Ft at 2 days after injection (a). Co‐labeling for anti‐Iba1 and L‐Ft at 5 days after injection (b). Perls' Prussian Blue staining at 2 days and 7 days after injection (c). Autofluorescence (AF) and immunolabeling for 8‐OHdG at 2 days after injection (d). AF and immunolabeling for carboxyethyl pyrrole (CEP) at 2 days (e) after injection. Co‐labeling for anti‐Iba1 and CEP at 5 days after injection (f). Co‐labeling for anti‐Iba1 and E06 at 5 days after injection (g). Co‐labeling for anti‐CD68 and MDA at 5 days after injection (h). Enlarged image of co‐labeling for Iba1 and CEP (white box, f1‐f3) (i). Enlarged image of co‐labeling for Iba1 and E06 (white box, g1‐g3) (j). Enlarged image of co‐labeling for CD68 and MDA (white box, h1‐h3) (k). White arrows indicate co‐labeling for GFAP and L‐Ft in a. Magenta arrows indicate immunolabeling for Iba1 and white arrows indicate immunolabeling for L‐Ft in b. Red arrows indicate Perls' staining, and black arrows indicate migrated RPEs in c. Yellow arrows indicate immunolabeling for 8‐OHdG in d; blue arrows indicate immunolabeling for Iba1 and CEP in e. Blue arrows indicate co‐labeling for anti‐Iba1 and CEP in f and i. Orange arrows indicate co‐labeling for anti‐Iba1 and E06 in g and j. White arrows indicate co‐labeling for anti‐CD68 and MDA in h and k. INL, inner nuclear layer; ONL, outer nuclear layer; RPE, retinal pigmented epithelium; CEP, carboxyethyl pyrrole; MDA, malondialdehyde. Representative images are shown from N = 3 mice per group. Scale bar: 50 µm

Immunolabeling for ferritin light chain (L‐Ft) and Perls' staining were conducted on cryosections to localize FAC‐induced iron accumulation in the retina. L‐Ft protein levels can be used as an indicator of iron, since L‐Ft levels are increased in response to elevated intracellular iron (Song et al., 2014). While Figure 2 showed FAC‐induced green and red emitting AF visible with long fluorescence microscopy exposure times, we used a shorter exposure time for all immunofluorescence imaging to avoid detecting the AF. No primary controls were used to verify that the secondary antibody was not binding non‐specifically and to show whether any AF was visible with the short exposure times. Co‐labeling of glial fibrillary acidic protein (GFAP) and L‐Ft was conducted on cryosections prepared at 2 days after injection. Müller cells overexpress GFAP in a gliotic response to many retinal injuries, pathological conditions, and aging. By 2 days after saline injection, L‐Ft weakly labeled the ganglion cell layer, outer plexiform layer, and inner segment layers (Figure 3a). In contrast, by 2 days after FAC injection, increased L‐Ft staining was observed in the ganglion cell layer, outer plexiform layer, and Müller cells co‐labeled with GFAP (Figure 3a, white arrows). By 5 days after injection, FAC increased L‐Ft labeling (white arrows) in Iba1‐labeled myeloid cells (magenta arrows) (Figure 3b). Perls' Prussian Blue staining for iron was positive within the neural retinal (Figure 3c, red arrows). RPE cell migration into the neural retina was observed (Figure 3c, black arrows). RPE hypertrophy and atrophy were evident (Figure 3c).

To assess FAC‐induced oxidative damage and lipid peroxidation, immunolabeling of 8‐hydroxy‐2′‐deoxyguanosine (8‐OHdG), carboxyethyl pyrrole, malondialdehyde, and oxidized phospholipids (E06 antibody) was conducted. 8‐OHdG is a DNA oxidation product. By 2 days after injection, 8‐OHdG labeling (yellow arrows) was found in RPE nuclei of FAC‐injected eyes, but not saline‐injected eyes (Figure 3d). By 2 days after FAC injection, increased immunolabeling for carboxyethyl pyrrole appeared in both photoreceptor inner and outer segments (Figure 3e, blue arrows); by 5 days after FAC injection, carboxyethyl pyrrole labeling was present in Iba1‐labeled myeloid cells and RPE cells (Figure 3f and i, blue arrows). The E06 antibody reacts with oxidized phospholipids. At 5 days, increased immunolabeling for E06 was present mainly in the RPE and Iba1‐labeled myeloid cells among the photoreceptor outer segments (Figure 3g and j, orange arrows). Immunolabeling for malondialdehyde showed no difference between FAC saline‐injected eyes at 2 days after injection (data not shown), but was present in CD68+ myeloid cells at 5 days after injection (Figure 3h and k, green arrows). Taken together, these results suggest IVT iron induced an accumulation of lipid peroxidation products, appearing first in the photoreceptors, then in subretinal myeloid cells and RPE cells, most likely due to phagocytosis of lipid peroxidation products in photoreceptor outer segments.

### 2.4 FAC injection induced acute myeloid cell infiltration in FAC‐injected eyes as well as chronic myeloid cell infiltration in fellow, saline‐injected eyes

**FIGURE 4:**
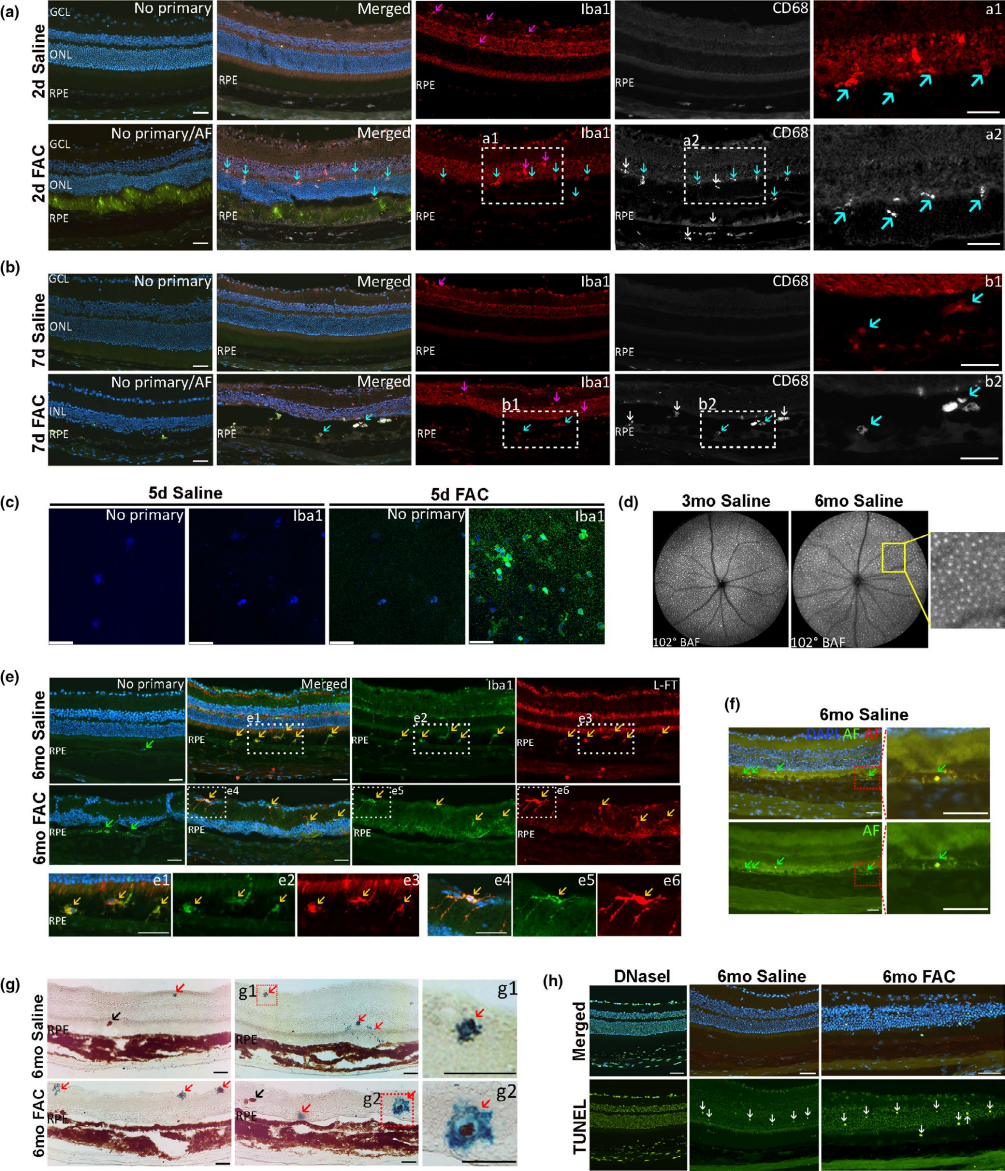
Ferric ammonium citrate (FAC) induced myeloid cell infiltration in FAC‐injected eyes and in saline‐injected fellow eyes of FAC‐injected eyes. Epifluorescence photomicrographs showing co‐labeling for Iba1 and CD68 on cryosections prepared on 2 days (a) and 7 days (b) after FAC or saline injection. Confocal imaging following immunolabeling for Iba1 was performed on retina flat mounts prepared at 5 days after FAC and saline injection (c). 102° ultra‐wide field blue‐AF cSLO images acquired at 3 months and 6 months after saline injection (d). Co‐labeling for Iba1 and L‐Ft on cryosections prepared at 6 months after injection (e). Autofluorescence (AF) on cryosections prepared at 6 months after saline injection (f). Perls' staining at 6 months after injection (g). TUNEL labeling at 6 months after injection (h). DNase I was used as a positive control for TUNEL labeling. White arrows indicate immunolabeling for CD68 in a and b. Magenta arrows indicate immunolabeling for Iba1, blue arrows indicate co‐labeling for Iba1 and CD68, and yellow arrows indicate co‐labeling for Iba1 and L‐Ft. Green arrows indicate AF in f. Red arrows indicate Perls' blue staining; black arrows indicate migrated RPEs in g. White arrows indicate TUNEL positive photoreceptors in h. BAF, blue‐AF. ONL, outer nuclear layer; RPE, retinal pigmented epithelium. Representative images are shown from N = 3 mice per group. Scale bar: 50 µm

At 7 days after FAC injection, autofluorescent cells were present between the neural retina and RPE layer. To determine whether some of the subretinal autofluorescent cells induced by FAC were myeloid cells (macrophages or microglia cells), co‐labeling with anti‐CD68 and anti‐Iba1 was performed; both label macrophages and microglia cells. At 2 days after saline injection, Iba1‐labeled microglial cells were present in the inner retina (Figure 4a, magenta arrows). At 2 days after FAC injection, Iba1+/CD68− cells (magenta arrows) were present in the inner nuclear layer and outer plexiform layer, and Iba1+/CD68+ cells (blue arrows) and Iba1−/CD68+ cells (white arrows) were present among the photoreceptor inner and outer segments (Figure 4a). Also, CD68 labeling was found uniformly in RPE cells within the RPE monolayer in FAC‐injected eyes. At 7 days, Iba1+/CD68− microglial cells (magenta arrows) were found in the inner retina, while both Iba1+/CD68+ cells (blue arrows) and Iba1−/CD68+ cells (white arrows) were present among the photoreceptor inner and outer segments, and exhibited AF (Figure 4b). Immunolabeling with anti‐Iba1 was conducted on retina flat mounts prepared at 5 days after injection. Iba1+ cells were present among the photoreceptor outer segments after FAC injection (Figure 4c).

**FIGURE 5:**
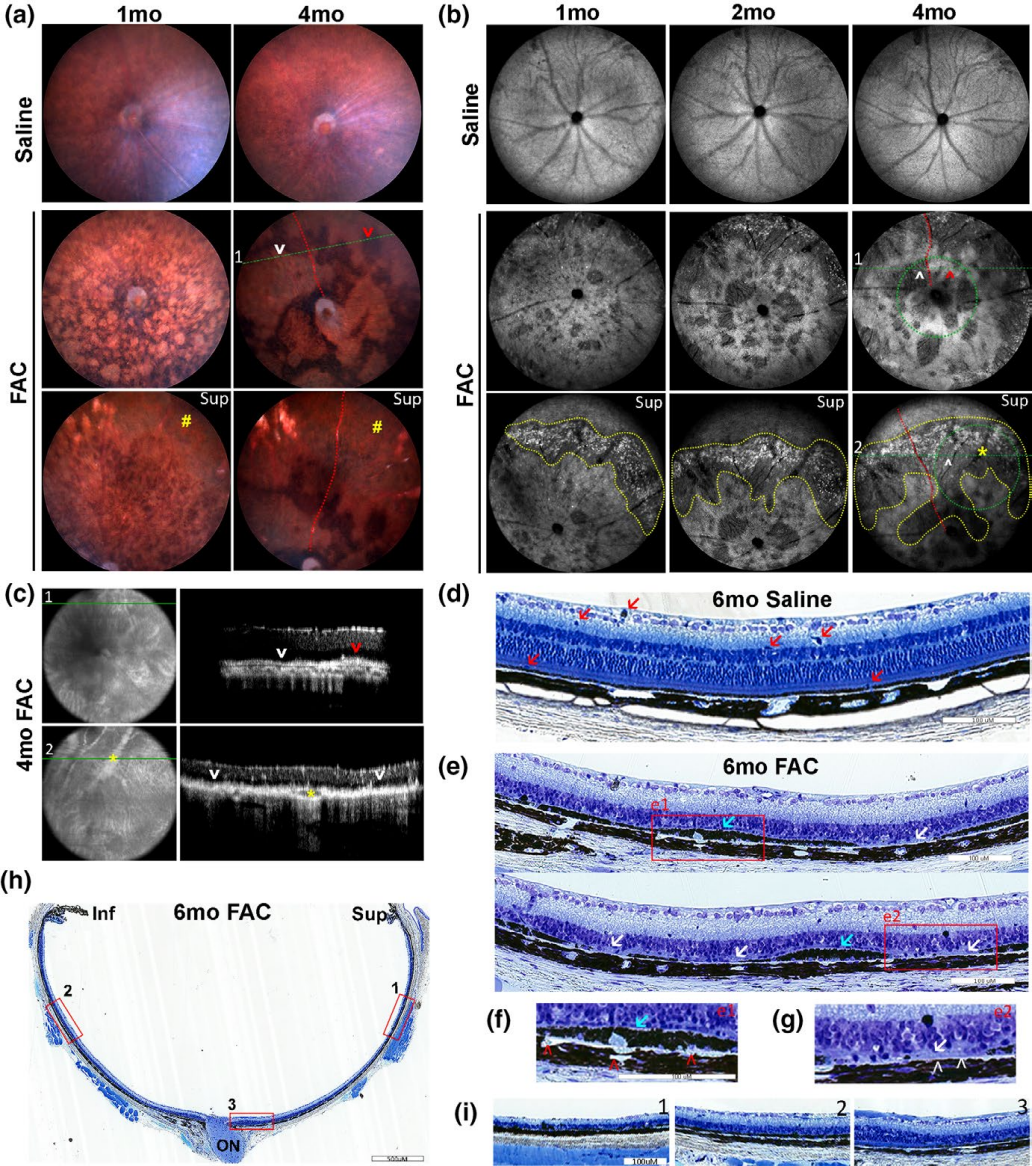
Ferric ammonium citrate (FAC) induces geographic atrophy with progression over time. Color fundus photographs obtained from the central retina and superior retina at 1 month and 4 months after saline and FAC injection (a). 102° ultra‐wide field infrared autofluorescence (AF) cSLO images obtained from central retina and superior retina at 1 month, 2 months, and 4 months after saline and FAC injection (b). OCT images from the same retina as in panel b in the central and peripheral retina (c). The positions of the line scans for c and d are indicated on the infrared AF cSLO images (b, green dotted lines). Toluidine blue staining conducted on plastic sections at 6 months after saline injection in the fellow eye (d) and on sections from the FAC‐injected eye (e‐i). Enlarged images from sections at 6 months after FAC injection (red boxes, e1 and e2) (f and g). Enlarged images from the superior peripheral retina (h, red box 1), central retina (h, red box 2), and inferior retina (h, red box 3) (i). Yellow pound signs indicate a geographic atrophy lesion in the superior retina in a. The yellow dotted lines demarcate a kidney bean‐shaped area geographic atrophy lesion in b; white arrowheads indicate atrophic RPEs, red arrowheads indicate residual RPEs, and asterisk indicates vortex vein. In e‐j, red arrows indicate myeloid cells, blue arrows indicate hypertrophic RPEs, white arrows indicate atrophic RPEs, red arrowheads indicate choriocapillaris, and white arrowheads indicate atrophy of choriocapillaris. ON: optic nerve; sup: superior retina; inf: inferior retina. Representative images are shown from N = 10 mice per group. Scale bar: 100 µm

At 3 and 6 months after FAC injection, infiltration of autofluorescent cells was observed in fellow eyes that had been injected with saline when the contralateral eye had been injected with FAC. These cells progressively accumulated over time, with abundant pan‐retinal autofluorescent cells by 3 months and 6 months (Figure 4d). This pattern was observed in all imaged mice, N = 10. In cryosections, co‐labeling with anti‐Iba1, L‐Ft, and AF imaging identified autofluorescent, iron‐laden myelocytes throughout the retinas of FAC‐injected and saline‐injected fellow eyes (Figure 4e,f). Consistent with this, Perls' Prussian blue staining labeled myeloid cells (red arrows) in both FAC‐injected and saline‐injected fellow eyes, as well as in pigmented RPE cells migrating into the neural retina (black arrows) (Figure 4g). TUNEL labeling was conducted to determine whether myeloid cell infiltration in saline‐injected fellow eyes may cause retinal damage. Both FAC‐ and saline‐injected eyes showed TUNEL labeling (Figure 5h, white arrows). Taken together, IVT FAC injection produced iron‐loaded myeloid cells. Several months after FAC injection, autofluorescent, iron‐loaded myeloid cells had infiltrated saline‐injected fellow eyes. In contrast, at 4 months after injection, mice receiving FAC in one eye and no injection in the other had no myeloid cell infiltration in the uninjected eye. Mice receiving saline injection in one eye and no injection in the other had no myeloid cell infiltration in either eye (data not shown).

### 2.5 FAC injection induced geographic atrophy onset and progression

At 1 month after FAC injection, color fundus photography showed extensive hypopigmentation in the central retina, and a continuous geographic atrophy geographic atrophy lesion developed in the superior retina by 4 months after injection (Figure 5a, yellow pound signs). Infrared images from the same eye were selected to display progression of the area of each geographic atrophy lesion at 1 month, 2 months, and 4 months after injection (Figure 5b). A kidney bean‐shaped geographic atrophy lesion initially developed in the superior retina at 1 month following FAC injection (demarcated by yellow dotted line) in all ten imaged FAC‐injected eyes. In the central retina, a few discontinuous atrophic lesions enlarged and fused together over time. RPE degeneration in the area that showed hypo‐AF in the infrared AF images was assessed by optical coherence tomography scans (55°) co‐aligned with the infrared AF (102°) images (green line), with retinal vessels (yellow asterisk, red dotted line) used as landmarks (Figure 5c). The hypo‐autofluorescent area in the infrared AF images (white arrowhead) corresponded to the lesion area with RPE atrophy (optical coherence tomography scans, white arrowhead) where light penetrated the choroid layer indicating an atrophic “window defect” in the RPE layer. The autofluorescent area in infrared AF images (red arrowhead) corresponded to an area with hyper‐reflective, persisting RPEs (red arrowhead) (Figure 5b,c). Toluidine blue staining conducted on plastic sections prepared at 6 months after FAC injection revealed myeloid cell infiltration in saline‐injected fellow eyes (Figure 5d, red arrows). In 6 months FAC‐injected eyes, photoreceptor degeneration, hypertrophic RPE (blue arrows), and atrophic RPE (white arrows) were observed (Figure 5e). A single layer of nuclei in the outer nuclear layer remained in the area with hypertrophic RPE while the outer nuclear layer was absent in the RPE atrophy area. The choriocapillaris was visible as a thin line beneath the RPE layer (Figure 5f, red arrowheads), containing RBCs. In contrast, choriocapillaris atrophy was found in the geographic atrophy area (Figure 5g, white arrowheads). A low magnification image displayed the retina thickness changes (Figure 5h). The superior retina showed more severe outer nuclear layer thinning than other regions (Figure 5i), and there was some preservation of the outer nuclear layer near the optic nerve head.

### 2.6 FAC injection induces chorioretinal neovascularization and atrophy

**FIGURE 6:**
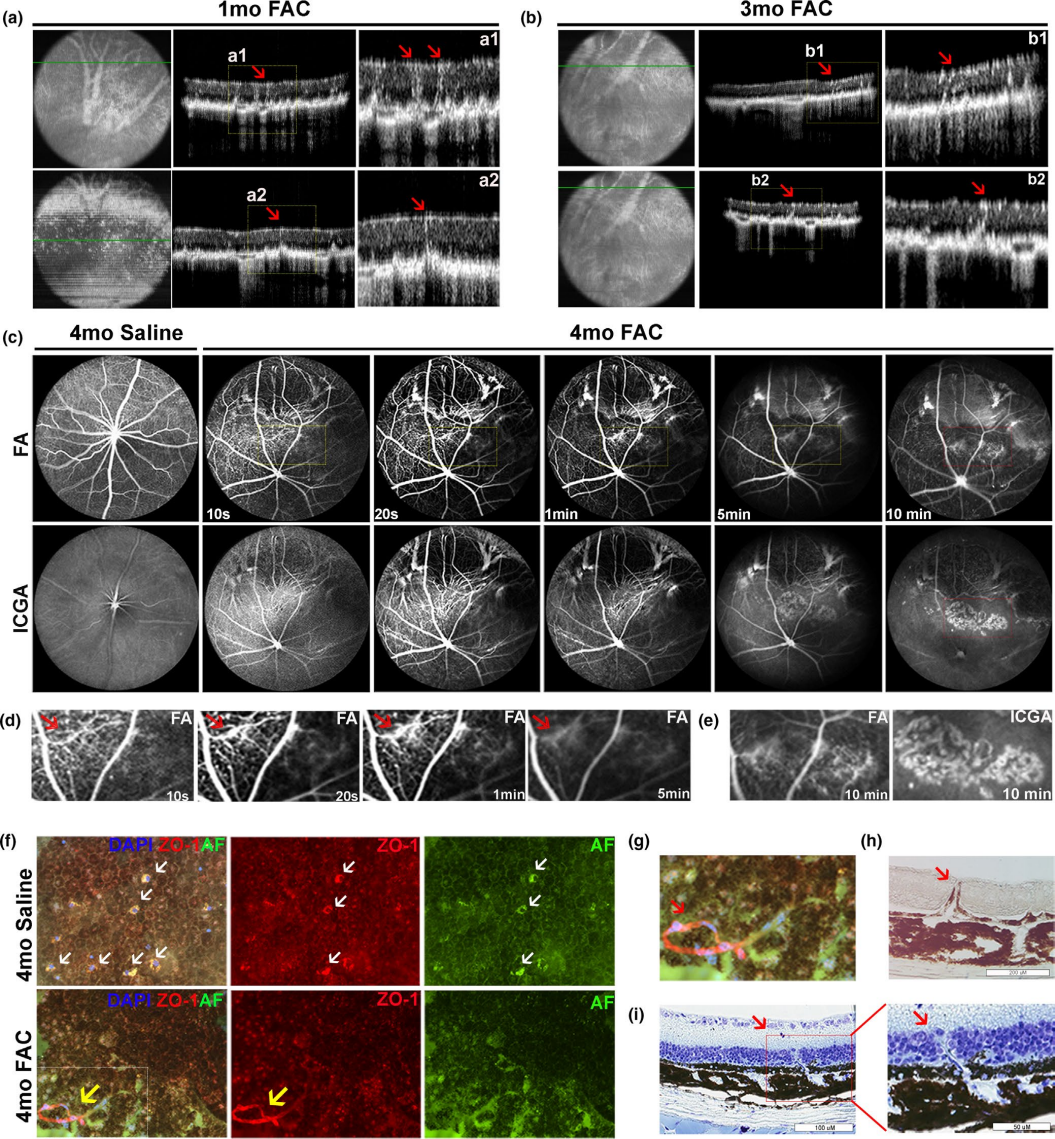
Ferric ammonium citrate (FAC) induces retinal and choroidal neovascularization. Optical coherence tomography images acquired at 1 month (a) and 3 months (b) after FAC injection. Simultaneous fluorescein angiography and indocyanine green angiography with cSLO imaging system at 4 months after FAC and saline injection (c‐e). Enlarged images of fluorescein angiography (c, yellow boxes) (d). Enlarged images of fluorescein angiography and indocyanine green angiography (c, red boxes) (e). Fluorescence imaging and immunolabeling for ZO‐1 conducted on RPE flat mount prepared at 4 months after injection (f). Enlarged images of autofluorescence (AF) and immunolabeling for ZO‐1 (f, white box) (g). Brightfield image of cryosection prepared at 6 months after FAC injection (h). Toluidine blue staining conducted on plastic sections prepared at 6 months after FAC injection (i). Red arrows indicate neovascularization. White arrows indicate infiltrated cells and yellow arrows indicate neovascularization in f. OCT, coherence tomography; FA, fluorescein angiography; ICGA, indocyanine green angiography. Representative in vivo images are shown from N = 10 mice per group. Representative histology images are shown from N = 3 mice per group

Optical coherence tomography scans showed neovascular lesions and RPE atrophy localized in the superior peripheral retina of FAC‐injected eyes by 1 month (Figure 6a, red arrows) and 3 months (Figure 6b, red arrows). The neovascular vessels extended from the choroid layer into the neural retina and had hypo‐reflective lumens (yellow boxes with red arrows, a1, a2, b1, and b2). Fundus fluorescein angiography and indocyanine green angiography at 4 months after FAC injection (Figure 6c‐e) showed fluorescence filling defects in the geographic atrophy area in the superior retina. Angiography also showed neovascularization; the 10 min indocyanine green image showed a vascular net at the level of the choroid (red box). Fluorescein leakage was seen in this area at 1 min after intravenous fluorescein injection (yellow box) (Figure 6c). Enlarged fluorescein angiography images from the central retina (yellow boxes) showed retinal vessels connected to the choroidal vascular net, suggesting a retinal angiomatous proliferation lesion (type III choroidal neovascular lesion) (Figure 6d). Late phase hyper‐fluorescence was observed in the central retina at 10 min on angiography, indicating fluorescence retention in neovascular lesions (Figure 6e).

Immunofluorescence imaging was conducted on RPE flat mounts labeled with ZO‐1. Infiltration of myeloid cells with DAPI labeled nuclei and red/green cytoplasmic AF was observed in the saline‐injected fellow eyes (white arrows); in FAC‐injected eyes, RPE cells had diminished ZO‐1 labeling, and neovascularization (yellow arrows) was observed (Figure 6f). Enlarged image (white box) showed neovascularization containing DAPI‐labeled vascular endothelial cell nuclei (Figure 6g). A choroidal neovascular lesion was evident in the brightfield image of cryosections, prepared at 6 months after FAC injection (Figure 6h, red arrows), and in the toluidine blue‐stained plastic sections at the same time point (Figure 6i, red arrows).

### 2.7 IVT FAC induced expression changes in antioxidant, iron‐regulating, and cell‐type‐specific genes in RPE and neural retina

To assess intracellular iron levels after FAC injection, mRNA levels of transferrin receptor (*Tfrc*) were measured by qPCR. *Tfrc* levels are inversely related to intracellular iron levels, so *Tfrc* measurement by qPCR is a reliable indicator of intracellular iron (Song et al., 2016). In the neural retina, at both 2 days and 7 days after FAC injection, *Tfrc* mRNA levels were significantly decreased. Levels were also decreased in the isolated RPE at 7 days but not yet at 2 days. To assess rod and cone stress and differentiation, mRNA levels of the rod‐specific gene rhodopsin (*Rho*), cone opsin1 medium wave sensitive and short wave sensitive (*Opn1mw and Opn1sw*) were measured. mRNA levels of *Rho*, *Opn1mw*, and *Opn1sw* were significantly decreased after FAC injection compared to saline at both time points (Figure S1a, d). Retinal pigment epithelium 65 (*Rpe65*) in RPE cells was quantified to detect RPE stress and differentiation. By 7 days, *Rpe65* was significantly decreased after FAC treatment compared to saline control (*p < 0.05, Figure S1f).

To investigate FAC‐induced oxidative stress, mRNA levels of antioxidants heme *oxygenase 1* (*Hmox1*), *catalase* (*Cat*), and *superoxide dismutase 1* (*Sod1*) were measured. *Hmox1*, *Cat*, and *Sod1* were significantly increased in FAC‐treated neural retina at both 2 days and 7 days, compared to saline (Figure S1b and e). In RPEs, the mRNA level of *Hmox1* was significantly upregulated at both 2 days and 7 days; however, both *Sod1* and *Cat* decreased at 2 days then recovered at 7 days, in FAC compared to saline. (**p < 0.01, Figure S1c and f). To investigate FAC‐induced inflammation, relative mRNA levels of *interleukin 1 beta* (*IL‐1β*), *interleukin 6* (*IL‐6*), *cluster of differentiation 68* (*Cd68*), and *complement C3* were detected by qPCR. *IL‐1β*, *IL‐6*, and *C3* were significantly increased in the neural retina at 2 days and 7 days (**p < 0.01, Figure S1b and e). In RPE cells, consistent with the immunofluorescence results, *Cd68* mRNA was significantly upregulated at 7 days, in IVT FAC compared with saline‐treated controls. (****p < 0.0001, Figure S1c and f).

To investigate whether FAC‐induced cell death was associated with changes in ferroptosis‐related genes, mRNA levels of *Gpx4* and *Slc7a11* were detected by qPCR in both neural retina and RPE cells. The mRNA level of *Gpx4* in FAC‐injected neural retina was significantly upregulated at 2 days compared to saline controls and significantly decreased at 7 days. *Slc7a11* was significantly increased in FAC‐treated neural retina at both 2 days and 7 days. In the RPE, there was no significant change to either of these genes (Figure S1a, c, d, and f).

## 3 DISCUSSION

This study revealed mechanisms of iron‐induced retinal toxicity as well as chronic effects of IVT FAC. We found temporal changes in the retinal AF spectrum, accumulation of carboxyethyl pyrrole and atRALdi and slowly progressive degeneration of the RPE, producing a new model of geographic atrophy. Surprisingly, we observed infiltration of iron‐laden myeloid cells into fellow eyes, which is in some ways similar to sympathetic ophthalmia, a disease that can affect fellow eyes of patients with ruptured globes.

In a prior study, we found that ferric sulfate was not acutely retina‐toxic. Herein, we found that equimolar ferric ammonium citrate (FAC) was toxic. The most likely explanation is that FAC is known to be more soluble than ferric sulfate (National Center for Biotechnology Information, 2021a, 2021b) and is reduced to toxic ferrous iron by the abundant (2 mM) ascorbate in the vitreous, which we confirmed in solution.

IVT FAC caused acute photoreceptor degeneration by 7 days. By 1 month after injection, RPE degeneration developed reproducibly (N = 10) into a large “kidney bean” shaped area of geographic atrophy specifically in the superior retina and several independent small geographic atrophy lesions throughout the retina. As in human geographic atrophy (Moschos et al., 2014), these atrophic areas expanded and fused over time. Mechanisms of RPE degeneration likely involve damage from toxic lipid peroxidation products and carbonyl‐carrying degradation products of bisretinoids. These products may impair RPE phago‐lysosomal processing. This may trigger lysosomogenesis, as we found an increase in the lysosomal marker Lamp1 in RPE lysates following IVT FAC (not shown).

IVT FAC also induced green‐emitting AF in mitochondria of the photoreceptor inner segments, and broad wavelength‐emitting AF in rod photoreceptor outer segments by 4 h after injection. There was also broad‐spectrum AF in myeloid and RPE cells at 7 days, presumably because these cells phagocytosed the AF material within degenerating photoreceptor outer segments. Previous studies of IVT iron reported an increase in broad‐spectrum AF in photoreceptors and RPEs (Dunaief, 2006; Shu et al., 2020). Katz et al. (1994) reported that IVT ferrous sulfate induced vitamin A dependent AF in photoreceptor outer segments and RPEs, consistent with the possibility that the AF results from bisretinoid formation. This is aligned with our finding of increased atRALdi at 7 days after IVT FAC.

The green‐emitting AF in mitochondria within photoreceptor inner segments may come from flavin adenine dinucleotide (FAD). Oxidized FAD is fluorescent, while reduced FADH_2_ is not (Heikal, 2010). FAD has fluorescence excitation at 450 nm and green emission maxima at 535 nm, which is consistent with the spectra observed by fluorescence microscopy in retinal cryosections of FAC‐treated eyes. Impaired reduction of FAD to FADH_2_ in the citric acid cycle could account for increased FAD fluorescence.

Bisretinoids are photosensitizers that can damage or kill the RPE. Here, we found IVT iron was associated with elevated atRALdi, a well‐characterized RPE lipofuscin bisretinoid. AtRAL di has been reported to be more abundant than A2E in the retinas from *Abca4*−/−*Rdh8*−/− mice, a model with features of recessive Stargardt disease (Zhao et al., 2017). Unconjugated atRALdi serves as a photosensitizer for the generation of singlet oxygen leading to oxidative damage; atRALdi is more susceptible to photooxidation than A2E (Kim et al., 2007). Elevated levels of atRALdi can induce apoptosis of cultured RPEs (Zhao et al., 2017).

Neuroinflammation and complement activation have been reported in AMD. Here, we report that IVT iron increased mRNA levels of *IL‐1β*, *IL‐6*, and complement factor C3 in the neural retina. Further, oxidative stress and Fenton reaction‐associated formation of carbonyl‐compounds from bisretinoid degradation can induce RPE cells to produce cytokines, including IL‐1, IL‐6, TNF‐α, and others, which recruit inflammatory cells including microglia and macrophages (Krizhanovsky et al., 2008). IVT iron induced Iba1+/CD68+ and Iba1−/CD68+ myeloid cell infiltration into the outer retina, consistent with increased mRNA levels of *CD68* in both neural retina and RPEs. These myeloid cells most likely consist of both blood‐borne macrophages and activated resident microglia. Consistent with this, Moos et al. (2011) reported macrophage infiltration into eyes injected with iron. Herein, there was also increased labeling for CD68 within the RPEs, and RPEs migration into the neural retina after iron injection. This mirrors observations from AMD patients where migrated RPEs were immunoreactive for CD68, suggesting transdifferentiation (Cao et al., 2020).

Surprisingly, we found progressive accumulation of autofluorescent cells in saline‐injected eyes when the fellow eye was injected with FAC. This pan‐retinal autofluorescent cell infiltration was observed by in vivo imaging beginning 2 months after the injections, with the number of infiltrating cells increasing over time. Immunohistochemistry revealed that these subretinal autofluorescent cells were Iba1+ indicating myeloid lineage. Unilateral IVT injection of iron or saline with no treatment in the fellow eye did not cause myeloid infiltration in the fellow eye. Saline injection in one eye did not cause myeloid infiltration in either eye. These results suggest that the myeloid cell infiltration in the saline‐injected fellow eyes of FAC‐injected eyes is a consequence of iron injection in the exciting eye combined with an intraocular injury in the fellow eye. Interestingly, there was iron deposition in the fellow eye myeloid cells, indicated by labeling with L‐Ft and Perls' staining. Some myeloid cells extended from the choriocapillaris to the subretinal space. Based on these data, we hypothesize that myeloid cells were recruited and activated in FAC‐injected eyes, and some of these migrated to the fellow eyes injured by the saline injection, similar to a sympathetic uveitis phenomenon.

In summary, the mechanisms of iron‐induced retinal toxicity were investigated in a mouse model with IVT injection of FAC. This is a novel model of iron‐induced acute and chronic retinal degeneration that shares multiple features with human AMD and other retinal diseases involving oxidative stress, lipid peroxidation, enhanced AF, inflammation, geographic atrophy, and choroidal neovascularization. This model will be useful for understanding ocular siderosis and testing retina‐protective therapies for a range of retinal diseases.

## 4 METHODS AND MATERIALS

### 4.1 Animals

Adult male wild‐type C57BL/6J mice (8 weeks, Stock No.000664; Jackson Labs) were housed in standard conditions under cyclic light (12 h:12 h light–dark cycle) with standard laboratory water and food available ad libitum. All housing and procedures were performed according to the NIH Guide for the Care and Use of Experimental Animals and approved by the University of Pennsylvania Animal Care and Use Committee.

### 4.2 Intravitreal injections

Intravitreal injections were performed as previously described (Hadziahmetovic et al., 2011). Eyes were injected with 1 μl of 0.5 mM FAC (MP Biomedicals LLC) diluted in 0.9% NaCl (saline) or 1 µl of saline as control.

### 4.3 In vivo imaging system

Mice were given general anesthesia and placed on a platform. Color fundus photograph and fundus AF were acquired using a Micron III fundus camera (Phoenix Research Laboratories, Inc). The Micron III fundus camera has a filter for color fundus photography between 450 and 680 nm; and blue light excitation filter between 440 and 485 nm. For visualization of retinal structure, spectral domain optical coherence tomography imaging was performed using a Bioptigen Envisu (R2200; Bioptigen Inc.) coupled to a broadband LED light source (T870‐HP; Superlum Diodes, Ltd.). Confocal scanning laser ophthalmoscopy (cSLO) (Spectralis HRA; Heidelberg Engineering) was employed for visualization of fundus AF using BluePeak™ or simply blue autofluorescence (488 nm excitation) and near‐infrared AF (787 nm excitation) imaging modes. Images of the central and peripheral retina were obtained using the wide‐field (55°) lens and ultra‐wide field (102°) lens. Simultaneous fluorescein angiography and indocyanine green angiography were performed for the visualization of retinal vasculature. Fifty microliters of fluorescein and indocyanine green mixture were injected intravenously; then, fundus images were acquired with the 102° ultra‐wide field lens.

### 4.4 Electroretinography

Mice were dark adapted overnight and then anesthetized. Pupils were dilated with 1% tropicamide saline solution (Akorn, Inc.). Two contact lens electrodes made of UV transparent plastic with embedded platinum wires were placed in electrical contact with the corneas. A platinum wire loop placed in the mouth served as the reference and the ground electrode. The electroretinograms were recorded with an Espion E3 system (Diagnosys LLC) with a ganzfeld Color Dome stimulator. The stage was positioned in such a way that the mouse's head was located inside the stimulator, thus ensuring uniform full‐field illumination. The flash intensities for recordings of rod a‐ and b‐waves were 500 and 0.01 scot cd m‐2 s delivered by the white xenon flash, and green (510‐nm maximum) LED, respectively. The cone b‐wave was elicited by 500 scot cd second m‐2 white xenon flash delivered on a rod‐suppressing steady green background of 30 scot cd m‐2. All electroretinography was performed at the same time of day.

### 4.5 Tissue preparation and immunofluorescence

Immunofluorescence was performed on 10 μm cryosections as described previously (Hadziahmetovic et al., 2011). Primary antibodies used: rabbit anti‐TOMM20 (1:200; Abcam); mouse anti‐CEP (1:200, a kind gift of John Crabb; Cleveland Clinic); mouse anti‐oxidized phospholipid (clone E06; 1:200; Avanti Polar Lipids); rabbit anti‐MDA (1:200; LSBio); rabbit anti‐Iba1 (1:1000; Wako); rat anti‐CD68 (1:200; Bid‐Rad); rabbit anti‐GFAP (1:200; Abcam); and rabbit anti L‐FT (1:1000; a kind gift of Maura Poli and Paolo Arosio, University of Brescia). Cy3 conjugated peanut agglutinin (PNA) (1:200; Novus Biologicals) was also used. Images were obtained with the epifluorescence microscopy (Nikon 80i microscope; Nikon) under two sets of exposure parameters. For AF imaging, we used a long exposure time (600 ms). For immunofluorescence, we utilized a short exposure time (50 ms). An exception to this was for anti‐Iba1, where different exposure times were required in each experiment, presumably due to different levels of Iba‐1 expression in the myeloid cell. The exposure times were as follows: Figure 3b: 150 ms, Figure 3f, g: 200 ms, Figure 4a: 60 ms, Figure 4b: 80 ms, and Figure 4e: 400 ms. Images were analyzed using NIS‐Elements (Nikon), and confocal microscopy (Leica TCS SP8 Confocal with STED 3×; Leica Microsystems Inc.). Excitation/emission filters for epifluorescence microscopy transmitted: 325–375/435–485 nm emission wavelength (used for DAPI); 450–490/500–550 nm (used for green‐emitting AF and Alexa Fluor 488 detection after immunofluorescence labeling); and 530–540 nm/590–650 nm (used for red AF and Cy3 detection after immunofluorescence labeling). The excitation/emission wavelengths for confocal microscopy were 405/412–458 nm (used for DAPI); 488/503–545 nm (used for green‐emitting AF and Alexa Fluor 488 detection after immunofluorescence labeling); and 550/562–632 nm (used for Cy3 detection after immunofluorescence labeling).

### 4.6 Bisretinoid analysis by HPLC

Eyes were enucleated and snap frozen on dry ice at 7 days after FAC or saline injection. Frozen eyes were processed as previously described (Sparrow et al., 2010). The extract was redissolved in chloroform/ methanol (2:1), and bisretinoids were measured by HPLC (Alliance system; Waters Corp.) (Kim et al., 2004). Absorbance peaks were identified by comparison to external standards. Molar quantities per eye were calculated from peak areas using standard concentrations determined spectrophotometrically together with published extinction coefficients. Values from each sample were calibrated to the number of eyes in a sample and were expressed as picomoles/eye.

### 4.7 TUNEL labeling

TUNEL labeling was performed on cryosections prepared as above as described previously using an In Situ Cell Death Detection Kit, Fluorescein (Roche) per the manufacturer's instructions (Shu et al., 2020).

### 4.8 Perls' Prussian blue and Toluidine blue staining

Four micrometer (4 μm) thick plastic sections were cut in the sagittal plane. Perls' staining was conducted on plastic sections to evaluate retinal iron levels as previously described (Theurl et al., 2016). Plastic sections were stained with toluidine blue to evaluate retinal morphology as previously described (Bhoiwala et al., 2015).

### 4.9 Neural retina flat mounts and RPE flat mounts

Eyes were enucleated and fixed in 4% PFA for 15 min. Cornea, lens, and retina were removed to make eyecups. Eyecups were incubated with primary antibody at 4°C overnight, rinsed with PBS three times, then incubated with secondary antibody for 1 h at room temperature, and rinsed again. Six radial anterior–posterior oriented scleral cuts were made, and a flower‐shaped neural retina flat mount or RPE/choroid flat mount was placed on a glass slide mounted with DAPI. Rabbit anti‐Iba1 (1:200) and donkey anti‐rabbit Alexa Flour 488 (1:200) were used for the neural retina flat mount. Rabbit anti ZO‐1 (1:200) and donkey anti‐rabbit Cy3 (1:200) were used for the RPE flat mount.

### 4.10 RNA extraction and Quantitative RT‐PCR

Neural retina and purified RPE cells were isolated as previously described (Hadziahmetovic et al., 2011). Gene expression changes in the neural retina and purified RPE cells were evaluated (Wolkow et al., 2012). *Gapdh* was used as an endogenous control. Taqman Probes (ABI) were used as follows: *Cat* (Mm00437992), *Cd68* (Mm03047343), *C3* (Mm00437838), *Hmox1* (Mm00516005), *IL‐1β* (Mm00434228), *IL‐6* (Mm00446190), *Gpx4* (Mm00515041), *Rho* (Mm00520345), *Rpe65* (Mm00504133), *Slc7a11* (Mm00442530), *Sod1* (Mm01700393), *Tfrc* (Mm00441941), *Opn1mw* (Mm00433560), and *Opn1sw* (Mm00432058). The amount of target mRNA was compared among the groups of interest. All reactions were performed in technical (3 reactions per eye) triplicates and biological replicates (3–5 mice per genotype).

### 4.11 Total iron and ferrous iron measurements

Stock solutions of 25 mM (di) ferric sulfate (Fe₂(SO_4_)_3_) and 50 mM ferric ammonium citrate (FAC) (C_6_H_8_FeNO_7_) were prepared in normal saline. Both stock solutions were vortexed for 30 s before use. 20 mM sodium L‐ascorbate normal saline solution was prepared. The 20 mM sodium L‐ascorbate solution was diluted 1:10 in PBS in two vials into which 10 μl of FAC or ferric sulfate stock solutions were added. The final volume of either sample solution was 10 ml and used in the iron assay after a 5 min incubation at 37°C. The level of total (ferrous and ferric) and ferrous iron was measured using an iron assay kit (MAK025; Sigma) according to the manufacturer's protocol. The final pH of the PBS‐diluted samples was 7.36 (25 μM ferric sulfate, 2 mM Sodium L‐ascorbate) and 7.43 (50 μM ferric ammonium citrate, 2 mM Sodium L‐ascorbate).

### 4.12 Statistical analysis

Mean ± SEM was calculated for each group. Student's, two‐tailed t test was used for the statistical analysis. All statistical analyses were performed using GraphPad Prism 6.0.

## CONFLICT OF INTEREST

The authors declare that the research was conducted in the absence of any commercial or financial relationships that could be construed as a potential conflict of interest.

## AUTHOR CONTRIBUTIONS

YL, BAB, YS, HJK, MG, KZ, and AR performed the experiments. YL and JLD analyzed data and drafted the manuscript. MP contributed essential reagents. All authors provided critical review of the manuscript. GS, JRS, and JLD provided funding for the study.

## Supporting information

Figure S1

## Data Availability

The data that support the findings of this study are available from the corresponding author upon reasonable request.
