# Supplementary figures and images for: Intraocular iron injection induces oxidative stress followed by elements of geographic atrophy and sympathetic ophthalmia
Source: Aging Cell. 2021 Oct 9;20(11):e13490. doi: 10.1111/acel.13490 (PMC8590099; doi:10.1111/acel.13490)

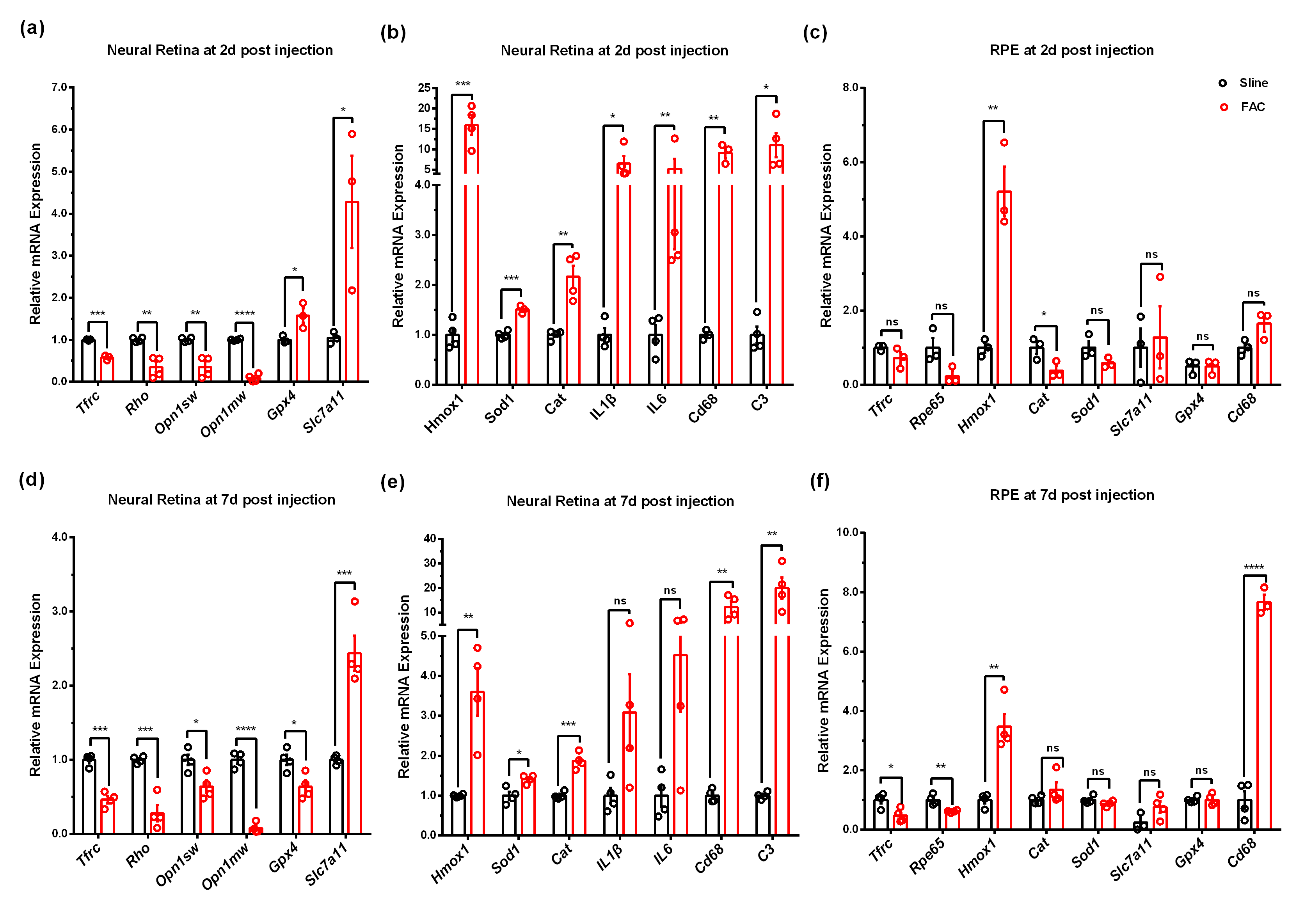

Supplement: Supplementary file 1 — Figure S1 [file ACEL-20-e13490-s001.png]
